# Opicapone to Treat Early Wearing‐off in Parkinson's Disease Patients: The European Experience

**DOI:** 10.1002/mdc3.70016

**Published:** 2025-02-19

**Authors:** Joaquim J. Ferreira, Werner Poewe, José‐Francisco Rocha, Olivier Rascol

**Affiliations:** ^1^ Laboratory of Clinical Pharmacology and Therapeutics, Faculdade de Medicina Universidade de Lisboa Lisbon Portugal; ^2^ Department of Neurology Medical University of Innsbruck Innsbruck Austria; ^3^ BIAL—Portela & C^a^ S.A. Coronado Portugal; ^4^ Departments of Neurosciences and Clinical Pharmacology and NS‐Park/FCRIN Network University of Toulouse 3, University Hospital of Toulouse, INSERM; Clinical Investigation Center CIC1436 Toulouse France

**Keywords:** COMT, levodopa, motor fluctuations, opicapone, wearing‐off

Dear Editors,

We read with interest the paper by Lee et al on their phase 4 clinical trial comparing the efficacy of adjunct opicapone 50 mg versus adding another levodopa (100 mg) dose^1^ in patients with PD and early signs of wearing‐off and wish to congratulate them on the successful execution of the South Korean component of the ADOPTION study program. Our parallel experience in Europe was hindered by very slow recruitment which led to the inclusion of an overall smaller sample size. This was due mainly to the difficulty to identify potential patients that were not already being treated with opicapone.

While no formal sample size calculation was performed for this exploratory trial, we planned to include 50 patients per arm. Following an extended recruitment of approximately 1.5 years and several recruitment drives, 106 patients were enrolled from 23 centers across Europe. Of those, 75 patients (71%) were randomized and treated with either opicapone 50 mg (*N* = 38) or levodopa 100 mg (*N* = 37). In the intent‐to‐treat population, we saw no significant differences between the opicapone and levodopa 100 mg groups. The LS mean (SE) change from baseline to end of study treatment in absolute *off* time was −64.4 min [17.6] in the opicapone 50 mg and −73.0 min [18.3] in the levodopa group, favoring levodopa treatment, while the responder analysis favored opicapone (66% of patients achieved ≥1 h *off* time reduction with opicapone versus 54% with levodopa 100 mg). However, post‐study checks identified that there was one non‐compliant site who had *only* recruited ineligible patients (*n* = 7, of whom 3 were randomized, raising a site data quality flag), as well as a number of participants enrolled at other sites who, despite meeting the broad inclusion criteria, exhibited atypical disease progression and/or extreme treatment responses that raised questions about the adequacy of their selection and/or assessment. For example, some participants had evidence of significant motor fluctuations within 6 months of diagnosis and levodopa initiation, while others reportedly had extremely large changes from baseline in OFF time. In the per protocol population, the adjusted mean change from baseline was −96.9 min [17.9] in the opicapone 50 mg group (*n* = 28) and −58.1 min [19.0] in the levodopa group (*n* = 25), and the treatment difference remained non‐significant, albeit more consistent with the South Korean findings. Results for other secondary outcomes also showed a non‐significant trend favoring adjunct opicapone.

The problems with the performance of some sites in our study highlights the risks of the pressure for recruitment and the importance of site and investigator selection and monitoring.^2^ The ADOPTION clinical program was purposefully designed to enable pooling of data from the similarly designed South Korean and European studies, and when pooled into a larger dataset, provided additional power to detect a significant treatment difference for adjunct opicapone versus an additional levodopa dose.^3^

## Author Roles

(1) Research Project: A. Conception, B. Organization, C. Execution; (2) Statistical analysis: A. Design, B. Execution; C. Review and critique; (3) Manuscript Preparation: A. Writing of the First Draft, B. Review and Critique.

J.J.F.: 1A, 1B, 1C, 2C, 3A.

W.P.: 1A, 1B, 1C, 2C, 3B.

J.F.R.: 1A, 1B, 1C, 2C, 3A.

O.R.: 1A, 1B, 1C, 2C, 3B.

## Disclosures

**Ethical Compliance Statement:** The clinical study protocol and the informed consent form were reviewed and approved by the respective independent ethics committees at each site. All patients included in the European ADOPTION study signed a patient consent form. We confirm that we have read the Journal's position on issues involved in ethical publication and affirm that this work is consistent with those guidelines.

**Funding Source and Conflicts of Interest:** The ADOPTION clinical program was funded by BIAL—Portela & Ca, S.A. JJF, WP and OR were members of the ADOPTION steering committee, and they or their institutions received fees for participation. JFR was employed by BIAL at the time of study.

**Financial Disclosures for the Previous 12 Months:** JJF has provided consultancy and received speaker fees from Lundbeck, BIAL, Biogen, Acadia, Abbvie, Sunovion Pharmaceuticals, Zambon, Affiris, Roche, ONO and SK Chemicals and has received grants from Abbvie, Bial, Medtronic and Angelini. WP has received lecture fees and honoraria for consultancy in relation to clinical drug development programs from Alterity, AbbVie, Affiris, AstraZeneca, Axovant, BIAL, Biogen, Britannia, Lilly, Lundbeck, NeuroDerm, Neurocrine, Denali Pharmaceuticals, Orion Pharma, Roche, Stada, Sunovion, Takeda, UCB and Zambon, as well as grant support from the MJFF and the EU FP7 & Horizon 2020 programs. J‐FR was employed by BIAL at the time of study. OR has participated in advisory boards and/or provided consultancy for AbbVie, Adamas, Acorda, Addex, AlzProtect, ApoPharma, AstraZeneca, Axovant, Bial, Biogen, Britannia, Buckwang, CereSpir, Clevexel, Denali, INC Research, IPMDS, Lundbeck, Lupin, Merck, MundiPharma, NeurATRIS, NeuroDerm, Novartis, ONO Pharma, Osmotica, Parexel, Pfizer, Prexton Therapeutics, Quintiles, Roche, Sanofi, Servier, Sunovion, Theranexus, Takeda, Teva, UCB, Vectura, Watermark Research, XenoPort, XO, Zambon; received grants from Agence Nationale de la Recherche (ANR), CHU de Toulouse, France‐Parkinson, INSERM‐DHOS Recherche Clinique Translationnelle, Michael J. Fox Foundation, Programme Hospitalier de Recherche Clinique, European Commission (FP7, H2020), Cure Parkinson UK; and received a grant to participate in a symposium and contribute to the review of an article by the International Parkinson and Movement Disorder Society.

## Data Availability

The data that support the findings of this study are available from the corresponding author upon reasonable request.
